# High, but variable prevalence of *Sarcocystis cruzi* infections in farm-raised American bison (*Bison bison*) beef destined for human consumption

**DOI:** 10.1186/s13071-025-06660-y

**Published:** 2025-02-01

**Authors:** Larissa S. de Araujo, Aditya Gupta, Marianne Dias Papadopoulos, Doaa Naguib, Jacquin Battle, Oliver Kwok, Asis Khan, Benjamin Rosenthal, Jitender P. Dubey

**Affiliations:** United States Department of Agriculture, Agricultural Research Service, Animal Parasitic Diseases Laboratory, Beltsville Agricultural Research Centre, Beltsville, MD 20705-2350 USA

**Keywords:** *Sarcocystis*, *Bison bison*, qPCR, Molecular, Prevalence, *Sarcocystis cruzi*

## Abstract

**Background:**

Bison (*Bison bison*) and cattle (*Bos taurus*) are closely related (can interbreed) and they also share many parasites. Cattle are commonly infected with one or more of the eight named *Sarcocystis* species: *Sarcocystis hirsuta, S. cruzi, S. hominis, S. bovifelis, S. heydorni, S. bovini*, *S. sigmoideus* and *S. rommeli*. Among these, the full life-cycle is known only for *S. cruzi. Sarcocystis cruzi* (transmitted via canids) is recognized as the most pathogenic *Sarcocystis* species, causing abortion, low milk yield and poor body growth. It has been experimentally cross-transmitted from cattle to bison and vice versa.

**Methods:**

We tested 200 bison tongues from three commercial sources (farms) (Nebraska #141; South Dakota #36; New Jersey and Pennsylvania #23). Frozen tongues were purchased and examined for *Sarcocystis* infection using light microscopy, histology and quantitative PCR (qPCR) targeting *18S* ribosomal DNA (*18S* rRNA) of *S. cruzi*. Lesions associated with degenerating sarcocysts were studied. The intensity of *Sarcocystis* infection in histological sections was quantitated.

**Results:**

*Sarcocystis cruzi-*like infections were detected in 129 of 141 (91.5%) tongues from Nebraska, 36 of 36 (100%) tongues from South Dakota and two of 23 (8.6%) tongues from New Jersey and Pennsylvania. Sarcocysts were detected in histological sections stained with hematoxylin and eosin in 167 of 200 samples. Light microscopy examination revealed that the sarcocysts had thin walls (< 1 µm thick) and appeared to be *S. cruzi*. However, in two samples, sarcocysts had thicker walls measuring up to 2.3 µm wide and 154 µm long and the sarcocyst wall was not striated; these two samples could not be characterized further. In three tongues, degenerating sarcocysts were recognized; two of these were associated with thick-walled sarcocysts. Molecularly, *S. cruzi* from bison was identical to that in cattle.

**Conclusions:**

In the present study of bison tongues, *S. cruzi* was the only species identified in bison using both molecular and morphological methods. An unidentified species of *Sarcocystis* found in two bison samples needs further study.

**Graphical abstract:**

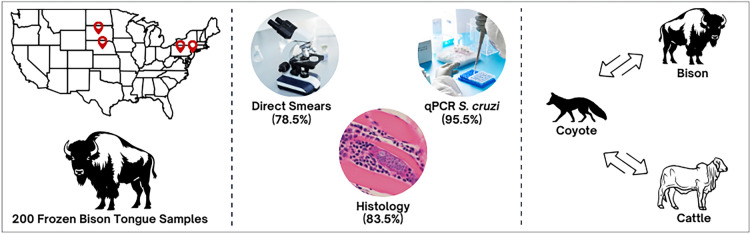

**Supplementary Information:**

The online version contains supplementary material available at 10.1186/s13071-025-06660-y.

## Background

American bison (*Bison bison*) is the biggest mammal in North America, and it was named the first national mammal of the USA in 2016 through the National Bison Legacy Act [1]. Historically, the American bison sustained the economy and societal well-being of indigenous peoples (Native Americans) in their native range. During the 1800s, millions of bison were slaughtered by European and American settlers, decimating the primary source of food and clothes as well as the culture of indigenous peoples via “The Great Slaughter” [2]. Bison hunting became a popular activity, and no protective measures were put in place; consequently, the animal was driven to near extinction [3].

According to the IUCN (International Union for Conservation of Nature and Natural Resources) Red List Assessment of 2017 [4], the American bison is listed as Near Threatened (NT). This listing is due to the small size of the wild bison population that is managed exclusively for conservation purposes, with most animals being managed for commercial purposes. The Red List shows the likelihood of a species becoming extinct in the near future; as such, the NT classification warns that American bison will soon likely qualify for the threatened category [4].

According to the National Agricultural Statistics Service (NASS) Census of Agriculture of 2022 published by the United States Department of Agriculture (USDA), the production and consumption of bison meat has increased within the past 5 years in the USA. The 2017 census shows that 52,937 bison on 1049 farms were managed for commercial purposes in 2017, increasing to 60,804 animals on 1201 farms in 2022, an increase of approximately 14.5%.

Bison meat has more protein and less fat than beef (USDA Agricultural Research Service: FoodData Central Food Data; 2018 [https://fdc.nal.usda.gov/fdc-app.html#/food-details/168608/nutrients]), and many consumers prefer the taste. American bison (*Bison bison*) and cattle (*Bos taurus*) are closely related (can interbreed) and share many common parasites. Cattle are commonly infected with one or more of the eight named* Sarcocystis* species: *Sarcocystis hirsuta*,* S. cruzi*,* S. hominis*,* S. bovifelis*,* S. heydorni*,* S. bovini*, *S. sigmoideus*, *S. rommeli*. Two* Sarcocystis* species*, S. hominis* and *S. heydorni*, are zoonotic [5, 6]. Of these, *S. cruzi* and *S. heydorni* form thin-walled (< 1 μm thick) sarcocysts whereas the remaining species have thick-walled (> 3 μm thick) sarcocysts. Among these, the full life-cycle is known for only *S. cruzi* (transmitted via canids); it is recognized as the most pathogenic species, causing abortion, low milk yield and poor body growth in cattle [7]. Thick-walled sarcocysts have been associated with abattoir condemnation due to infected meat [8].

Nothing is known of the prevalence of *Sarcocystis* spp. in the American bison raised for human consumption. Four decades ago, thin-walled sarcocysts were reported in the muscles of two of 15 (13%) feral bison from the National Bison Range, near Missoula, Montana [9]. Thin-walled sarcocysts were also found in sections of muscles of a bison that was found dead near Livingston, Montana; a laboratory-raised coyote excreted *S. cruzi* sporocysts in its feces after feeding on the muscles of this bison [10]. In another experiment, thin-walled sarcocysts were detected in sections of the esophagus and heart of a hunter-killed bison from Montana; 11 days after feeding on the infected parts, a laboratory-raised coyote (#13) excreted *S. cruzi*-like sporocysts [11]. In a subsequent study, *S. cruzi* from cattle (*Bos taurus*) was transmitted successfully to laboratory-raised bison and *S. cruzi* from bison was transmitted to cattle [12]. In this study, two 1-week-old bison were fed *S. cruzi* sporocysts from the feces of a laboratory-raised coyote that had been fed *S. cruzi*-infected meat of a cow (*Bos taurus*) from Montana [12]. One bison was fed 10 million sporocysts and developed acute sarcocystosis; the second bison was fed 100,000 sporocysts and developed muscle sarcocysts. Additionally, three cattle calves inoculated with 100,000 or 700,000 sporocysts from coyote #13 developed clinical *Sarcocystis* infections [13]. Two calves had diarrhea on 19–25 days post-inoculation (dpi); one of the two calves fed 700, 000 sporocysts became moribund on 28 dpi. Although limited, data from this study indicated that the bison strain of *S. cruzi* was more pathogenic than the cattle strain of *S. cruzi* [13] and that clinical signs might vary with host species. The diarrhea observed in one bison and three experimentally infected calves was unusual because it was normally not observed in cattle fed the bovine isolate of *S. cruzi* [13].

Here, we report the first survey of *Sarcocystis* infections in commercially raised bison in the USA.

## Methods

### Naturally infected bison

Two hundred frozen bison tongues were purchased from three farms located in different states (Nebraska #141, South Dakota #36, New Jersey and Pennsylvania #23) between October 2023 and March 2024 (Table 1). Bison from Nebraska and South Dakota were raised on open pasture and were 100% grass-fed; bison from New Jersey and Pennsylvania (1 owner) were raised on semi-open range and fed primarily on grass, enriched with a mix of minerals and grains as a supplement.

**Table 1:** Positivity of bison tongue samples for *Sarcocystis* spp. separated by source and methodology

Source	Number of samples	Positivity (no. counts and percentage)		
		Compression smears	Histology	qPCR for *Sarcocystis cruzi*
Nebraska	141	121/141 (85.8%)	129/141 (91.4%)	140/141 (99.2%)
South Dakota	36	35/36 (97.2%)	36/36 (100%)	36/36 (100%)
New Jersey and Pennsylvania	23	½3 (4%)	2/23 (8%)	15/23 (65.2%)
Total	200	157/200 (78.5%)	167/200 (83.5%)	191/200 (95.5%)

*qPCR* Quantitative PCR

### Testing for *Sarcocystis* infection

All samples were examined microscopically using several procedures.

#### Microscopic examination of muscle by the compression method

Small pieces (approx. 2 × 2 cm) of each tongue were compressed between a glass slide and coverslip and examined microscopically for sarcocysts. A sample was considered to be negative for *Sarcocystis* infection when no sarcocysts was detected in any of 30 preparations.

#### Histological examination

Four or more pieces (enough to fill a paraffin 4 × 2-cm cassette) of each tongue were fixed in 10% buffered formalin and processed for histology using paraffin embedding. Three 5-μm-thick sections were stained with hematoxylin and eosin (HE) and examined microscopically.

### Tongues stored in freezer

Approximately 50 g of each tongue was stored at - 80 °C for future study.

### Photographs

Cysts were photographed using a digital DP73 camera (Olympus Optical Ltd., Tokyo, Japan) attached to an Olympus AX 70 microscope (Olympus Optical Ltd.).

### DNA isolation and amplification

Each tongue muscle (50 g) was homogenized with 250 ml of 0.85% aqueous NaCl (saline) in a blender and passed through a cheesecloth; part of the filtrate was collected in a 50-m; tube and centrifuged for 10 min at 400 *g* [5]. The supernatant was discarded and the pellet resuspended with 2.5 ml of 0.85% NaCl (saline); a portion of each suspended pellet was processed for DNA extraction. For this, part of the homogenization product was transferred to a 2-ml microtube and centrifuged; the supernatant was discarded and the pellet was stored at - 80 °C. Part of the homogenization product was transferred to a 2-ml microtube and centrifuged again in a microtube centrifuge for 10 min at 100 × *g*. The supernatant was discarded, and the pellet was extracted using the QIAGEN DNeasy® Blood and Tissue Kit (QIAGEN, Hilden, Germany) utilizing a modification of the manufacturer’s protocol (the lysis step was carried out at 56 °C overnight in a dry bath incubator on an Eppendorf® ThermoMixer F1.5 [Eppendorf, Hamburg, Germany] and DNA samples were eluted in 100 μl of QIAGEN® Nuclease Free Water [QIAGEN] during last step). Extracted DNA samples were quantified and assessed using the NanoDrop® 1000 full-spectrum spectrophotometer (V3.8; Thermo Fisher Scientific, Waltham, MA, USA) and kept at - 20 °C until further analysis.

### Quantitative analysis

We performed quantitative real-time PCR (qPCR) targeting the* 18S* ribosomal RNA (*18S* rRNA) subunit of *S. cruzi* in the first four samples, employing four dilutions (10, 20, 40 and 60 ng) compared to non-diluted samples. This validated the use of stocks diluted to 10 ng. Those few samples estimated by spectrophotometry (NanoDrop® 1000 system) to contain < 10 ng/μl were concentrated using a SpeedVac Vacuum Concentrator (Thermo Fisher Scientific).

The qPCR tests targeted the *18S* rRNA gene marker of *S. cruzi* using qPCR probes. Forward and reverse primers and a probe were designed using Primer3 [14] by incorporating the *S. cruzi* reference sequence (GenBank accession number: KT901173). The primers and probe designed were forward primer q_*S. cruzi *(5’-ATA GTC ATA TCA GAT GAA AAT CTA C-3’), reverse primer q_*S. cruzi* (5’–CAG CCA TAT AAA ATG ACC ATA-3’) and probe q_*S. cruzi* (5’–ATC TGT TAA CAG CAG GTG GTG TAA AAA AGG T/3BHQ–3’). The PCR reactions were carried out using the Applied Biosystems™ QuantStudio 7 Flex Real-Time PCR System 7, and 96-well plates were used in addition to the IDT PrimeTime Gene Expression Master Mix Protocol (Integrated DNA Technologies, Inc., Coralville, IA, USA). The PCR tests were carried out in a total reaction volume of 20 μl, containing 10 μl PrimeTime MasterMix 2×, 0.5 μl forward primer, 0.5 μl reverse primer, 0.3 μl probe, 6.7 μl molecular biology grade water and 2 μl DNA) (Additional file: Table S1).

Negative samples according to the qPCR results were submitted to a second run using 60 ng (6 μl DNA) and the same qPCR conditions, with changes only in the quantity of DNA and molecular biology grade water. Accordingly, PCR tests were carried out in a total reaction volume of 20 μl, containing 10 μl PrimeTime MasterMix 2×, 0.5 μl forward primer, 0.5 μl reverse primer, 0.3 μl probe, 2.7 μl molecular biology grade water and 6 μl DNA.

### Polymerase chain reaction

Based on the cycle threshold (CT) values and qPCR results and comparing these with the light microscopy (LM) results, we selected nine bison tongue samples (sample numbers: 8, 94, 108, 148, 165, 181, 191, 193 and 199) for conventional PCR testing using specific primers targeting the *18S*, *28S* (*28S* RNA) and cytochrome* c* oxidase subunit 1 (*cox1*) regions of *Sarcocystis* species.

The 25-µl PCR mix reaction volume consisted of 2 μl of DNA template, 12.5 μl of Platinum Hot Start PCR Master Mix (Invitrogen, Thermo Fisher Scientific), 1 μl of 10 pmol/μl of each primer (Integrated DNA Technologies, Inc.) (Table 2) and 8.5 μl of molecular grade water. The PCR cycling parameters consisted of an initial denaturation at 94 °C for 3 min, followed by 35 cycles of denaturation at 94 °C for 30 s, annealing at 60 °C for 30 s and elongation at 68 °C for 20 min, with a final elongation of the incubated products at 68 °C for 5 min. In the case of negative results or low amplifications, this first PCR round was followed by a second round of PCR using the amplified products. All PCR products were analyzed by electrophoresis in a 2% agarose gel, and size was estimated by comparison with the 100-bp Plus DNA Ladder followed by DNA cleaning [15]. The final purified PCR products were sent for sequencing to the DNA sequencing Unit of Psomagen (Rockville, MD, USA), a commercial sequencing company for direct sequencing on an ABI 3500xl Genetic Analyzer (Applied Biosystems™, Thermo Fisher Scientific) using the primer sets specially designed for this study to obtain both strand reads. The sequences were deposited in the GenBank database, and the accession numbers were obtained (Table 2).

**Table 2:** Accession numbers and PCR primers used for the amplification and sequencing of different gene markers for *Sarcocystis cruzi*

Sample number	Gene^a^	Bases amplified (*n*)	PCR amplification primers (5’-3’)^b^	Sequencing primers (5’-3’)^c^	Accession number
8	*18S*	670	47F × 1462R	472F, 818F	PQ285147
94	*18S*	924	47F × 1462R	472F, 818F	PQ285149
	*28S*	845	28F × 943R	28F, 421R	PQ285158
	*cox1*	917	347F × 636R	77R, 636F, 401R, 94F	PQ427140
108	*18S*	670	47F × 1462R	472F, 818F	PQ285153
	*cox1*	852	347F × 636R	77R, 636F, 401R, 94F	PQ427141
148	*18S*	1342	47F × 1462R	472F, 818F, 1462R	PQ285154
	*28S*	843	28F × 943R	28F, 421R	PQ285160
	*cox1*	913	347F × 636R	77R, 636F, 401R, 94F	PQ427142
165	*18S*	1360	47F × 1462R	472F, 818F, 1462R	PQ285155
	*cox1*	947	347F × 636R	77R, 636F, 401R, 94F	PQ427143
191	*18S*	1387	47F × 1462R	472F, 818F, 1462R	PQ285156
	*cox1*	608	347F × 636R	77R, 636F, 401R	PQ427144
193	*18S*	675	47F × 1462R	472F, 818F	PQ285157
199	*18S*	964	47F × 1462R	472F, 818F, 1462R	PQ285159
	*cox1*	357	347F × 636R	347F, 636R	PQ427145

*F* Forward,* R* reverse

^a^*cox1* Cytochrome* c* oxidase subunit 1,* 18S/28S* 18S/28S ribosomal RNA subunits

^b^47F, GCCATGCATGTCTAAGTATAAGC; 1462R, ACACGCAAAGTCCCTCTAAGA; 28F, AGCGGTGGAAGAGAAAATAACA; 943R, ATAGATCATGGTCGGTCGTAAG; 347F, TTTCGGATGCGGTTCGCTAT; 636R, GTGCCTCCCAGGCTGAATAG

^c^818F, AGAGTGTTTGAAGCAGGCTAA; 421R, TCCAATCGCTTCCTTTTCAGC; 77R, AGACCGTGGAGCGTAAACAG; 636F, GGAGGCCGTTGACTGGATAG; 401R, CAGGATGGTGCCCAGGAAAT; 94F, CGGAGGAGATGCCGTTCTTT

### Phylogenetic reconstructions

Amplifications were attempted for all nine bison samples using *18S* rRNA and mitochondrial *cox1* genes. We also succeeded in amplifying *28S* rRNA from two bison samples (#94 and #148). The resulting sequences were trimmed and aligned by MAFT alignment (using the L-INS-i algorithm and 1.53 gap open penalty) in Geneious Prime, followed by ClustalW multiple alignment in MEGAX [16]. Any ambiguous bases were clarified by checking the respective chromatograms. The consensus sequences were then analyzed using a standard online Basic Local Alignment Search Tool (BLAST) (https://blast.ncbi.nlm.nih.gov/Blast.cgi) [17] against the genetic dataset available at the National Center for Biotechnology Information (NCBI).

The web server GUIDANCE2 [18] was used to align and remove ambiguously aligned positions. Specifically, the sequences were aligned with the MAFFT algorithm under the options Max-Iterate: 1000 and Pairwise Alignment Method: –local pair. Positions with a score < 0.93 were removed. Phylogenetic relationships were reconstructed under the maximum likelihood (ML) criterion. ML analyses were performed with the program IQ-TREE version 1.6.12 [19]. The analyses were run with the options –m MFP –b 1000. All codon positions were used. The models selected based on the Bayesian information criterion (BIC) criterion were JC + G + I and K2 + G + I for *18S* and *cox1*, respectively.

## Results

### Microscopy

**Fig. 1:**
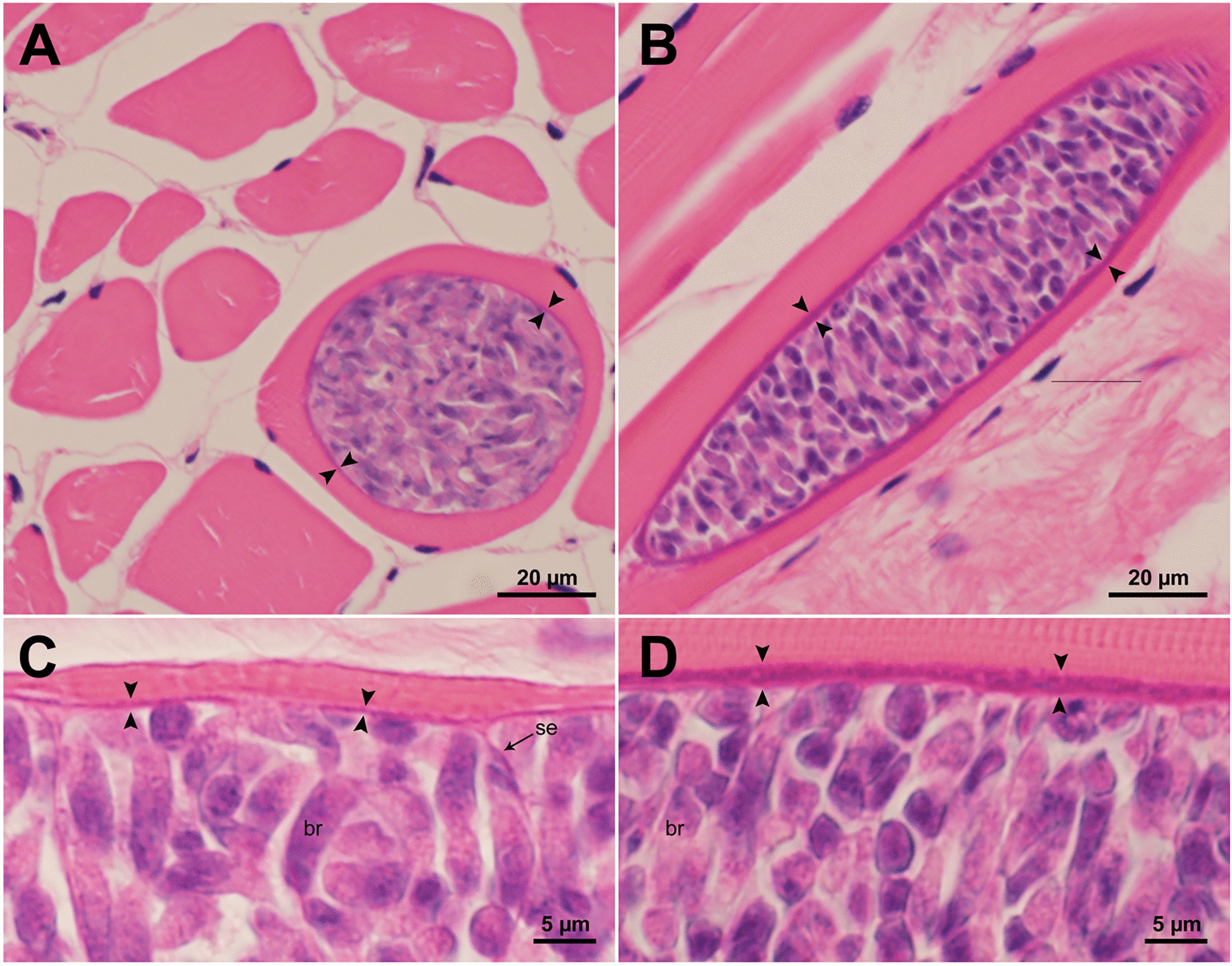
Comparison of thin-walled and thick-walled sarcocysts detected in histological sections (hematoxylin and eosin staining) of the tongue of bison #94 from Nebraska. Arrowheads point to wall thickness. **A** Thin-walled *Sarcocystis cruzi* sarcocyst at low resolution. **B** Unidentified sarcocyst with relatively thicker sarcocyst wall at low resolution. **C** *Sarcocystis cruzi* sarcocyst at higher resolution; note thin septa (se) and robust bradyzoites (br); **D** Unidentified sarcocyst with relatively thicker cyst wall at higher resolution; note robust bradyzoites (br)

Microscopic examination of compression preparations detected the presence of sarcocysts in 157 of 200 (78.5%) samples (Table 1); following staining with HE, sarcocysts were detected in 167 of 200 the histological sections (Table 1). The sarcocysts had thin walls (< 1 um thick) and appeared to be *S. cruzi* (Fig. 1a, c). However, in two bison samples (sample #94 and #108), sarcocysts had thicker walls measuring up to 2.3 μm wide and 154 μm long (Fig. 1b, d).

The number of sarcocysts per histologically stained slide ranged from 0 to 260 (Table 3). No sarcocysts were seen in 33 tongues, and 50 tongues had fewer than six cysts.

**Table 3:** Intensity of sarcocysts in 2 × 4-cm sections of bison tongues on hematoxylin and eosin-stained slides

Number of sarcocysts	Total* n* bison samples
0	33
1–6	50
7–12	31
13–18	26
19–24	15
25–30	13
31–36	9
37–42	2
43–48	5
49–54	3
55–60	2
61–66	2
67–72	2
85–90	1
97–102	2
121–126	2
145–150	1
169–174	1
259–264	1

**Fig. 2:**
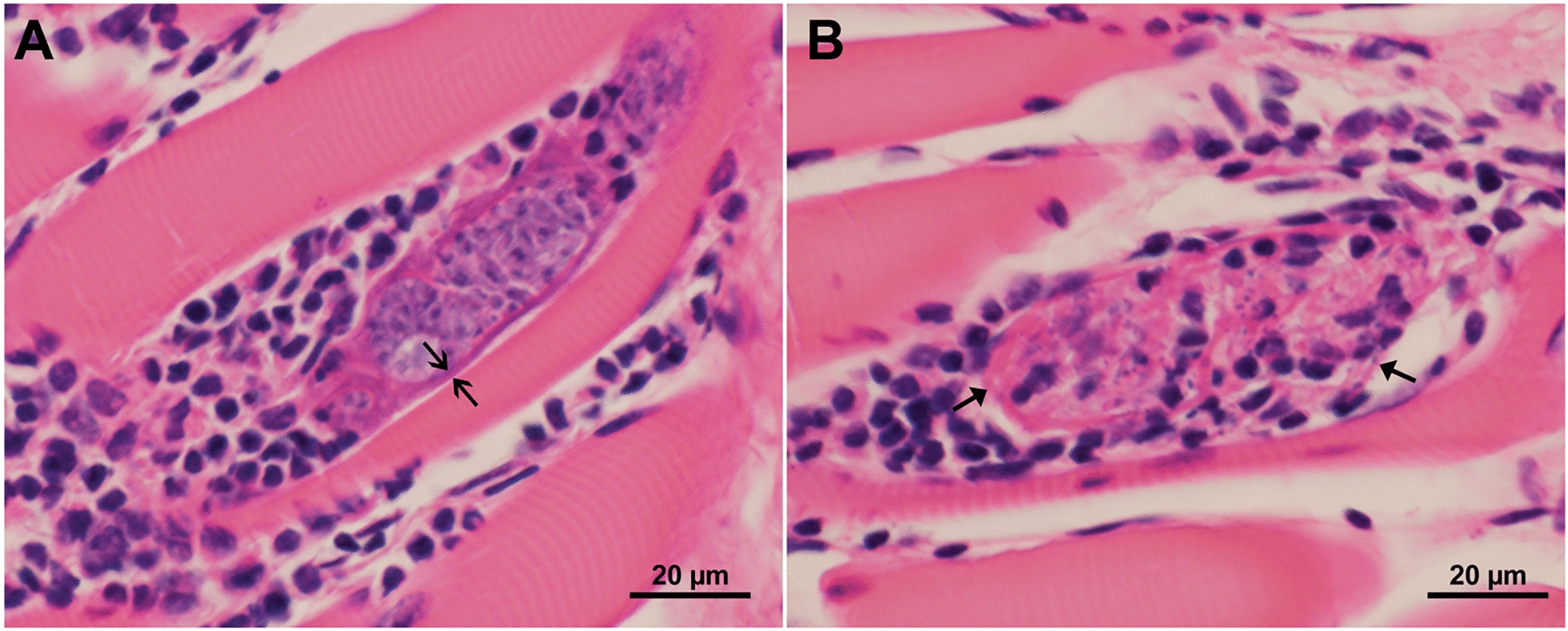
Myositis in the tongue of bison #94 from Nebraska. Hematoxylin and eosin staining. **A** A partly degenerating sarcocyst with mononuclear cell infiltration; note thick sarcocyst wall (arrow). **B** A focus of mononuclear infiltration around and inside a degenerating sarcocyst; the sarcocyst wall (arrow) is still visible

Mononuclear cell infiltrations were detected in 22 tongues (Fig. 2). Degenerating sarcocysts were recognizable in three tongues, of which two (sample #94, #108) were associated with thick-walled sarcocysts. In some of the degenerating sarcocysts, the cyst wall was apparent, whereas in others no cyst wall was recognizable.

### Molecular analysis

Quantitative PCR using *18S* rRNA for *S. cruzi* revealed 95.5% positivity in all bison tongue samples (140/141 samples from Nebraska, 36/36 from South Dakota and 15/23 from New Jersey and Pennsylvania) (Table 1).

Based on the results from all methods, we determined that the prevalence of *Sarcocystis* spp. in bison tongue samples was 95.5% (191/200) (Table 1). Of the 141 tongue samples from the Nebraska bison, *Sarcocystis* infections were detected in 140. Sample #8 from Nebraska, assessed to be positive for *Sarcocystis* spp. based on histology showing a thin-walled sarcocyst, tested negative for *S. cruzi* by qPCR, indicating the possible presence of a distinct species of *Sarcocystis*. Samples #94 and #108 were positive for *S. cruzi* and, both thin-walled and thick-walled cysts were detected, suggesting a co-infection with a different *Sarcocystis* spp. whose identity could be determined from the methods employed in the study.

Of the nine samples studied using PCR, sample #181 did not amplify after many rounds of amplifications. Samples from South Dakota were all positive for *S. cruzi* based on the results of each of the three diagnostic methods, while infections were less prevalent in samples from New Jersey and Pennsylvania. Additionally, positive samples from New Jersey and Pennsylvania showed a higher CT value, indicating a low infectivity, which correlates with the results (Additional file 1: Table S1).

Sanger sequencing of the *18S* and *cox1* genes corresponded well with the qPCR results and showed 99% identity to the published *S. cruzi* sequences. Analyses of the *18S* and *cox1* genes of *Sarcocystis* also revealed only a few differences between the obtained sequences and those isolated from wood bison (*Bison bison athabascae*) [20] and European bison (*Bison bonasus*) [21] (Additional file 2: Figure S1, Additional file 3: Figure S2).

For constructing consensus trees, we first blasted the edited sequences against the NCBI nucleotide database (https://www.ncbi.nlm.nih.gov/nucleotide/) and then downloaded the most closely related *Sarcocystis* species. All of the haplotypes used for the tree showed > 95% homogeneity with the sequence we obtained from bison samples #94 and #108. The analysis was performed against the existing partial sequences previously obtained from different isolates of *S. cruzi* and closely related *Sarcocystis* spp. Isolates of *S. bovifelis* were used as outgroups. The final dataset included 16 taxa and 1465 positions for *18S* and 16 taxa and 992 positions for *cox1*.

During the study, apart from interspecific variations, which ranged from 95% to 99% between different isolates of *S. cruzi* obtained from the different locations and hosts, intraspecific variations (> 99%) were also found between the sequences of different bison tongue samples isolated during the study.

## Discussion

*Sarcocystis cruzi* is one of the most prevalent microbial infections in cattle worldwide. In some surveys, nearly 100% of cattle have been found to be infected, as previously summarized [5, 8]. Several factors explain the highly efficient transmission of this infection: (1) several canids (dogs, coyote, foxes, wolves) can excrete millions of *Sarcocystis* sporocysts in feces; (2) sporocysts are sporulated and excreted in a fully infective stage; (3) sporocysts are environmentally resistant; and (4) canids can excrete sporocysts for months after a single infective meal [5]. The number of sarcocysts in cattle muscle varies, but to our knowledge, no published record exists. Although *S. cruzi* can be transplanted transplacentally in cattle, this transmission route is rare, and infected fetuses probably die in utero [5].

From a transmission perspective, the ingestion of only a few sarcocysts by a canid can lead to the excretion of many sporocysts. It should be noted that a bison can weigh up to 1000 kg, and that our estimates of sarcocyst numbers were derived from small sections of muscle on a histology slide (approx. 10 mg of tissue). In this study, we found up to 264 sarcocysts on one histology slide (Table 3).

The very high prevalence of *Sarcocystis* infections in the South Dakota and Nebraska samples compared with a lower prevalence in the New Jersey and Pennsylvania samples is probably related to management. The semi-open range system, its enriched diet and/or reduced contact with carnivores in the East of the USA may have protected the Eastern herds sampled here. Smaller populations of coyotes and the presence of Eastern Coyotes (*Canis latrans* var.), which are a canine hybrid of coyotes and wolves, might interfere with the passage of the parasite in the Eastern USA. Recently, wolves in Minnesota were found to be the definitive host for *S. cruzi* [22]. Wolves are rare in the Eastern USA.

**Fig. 3:**
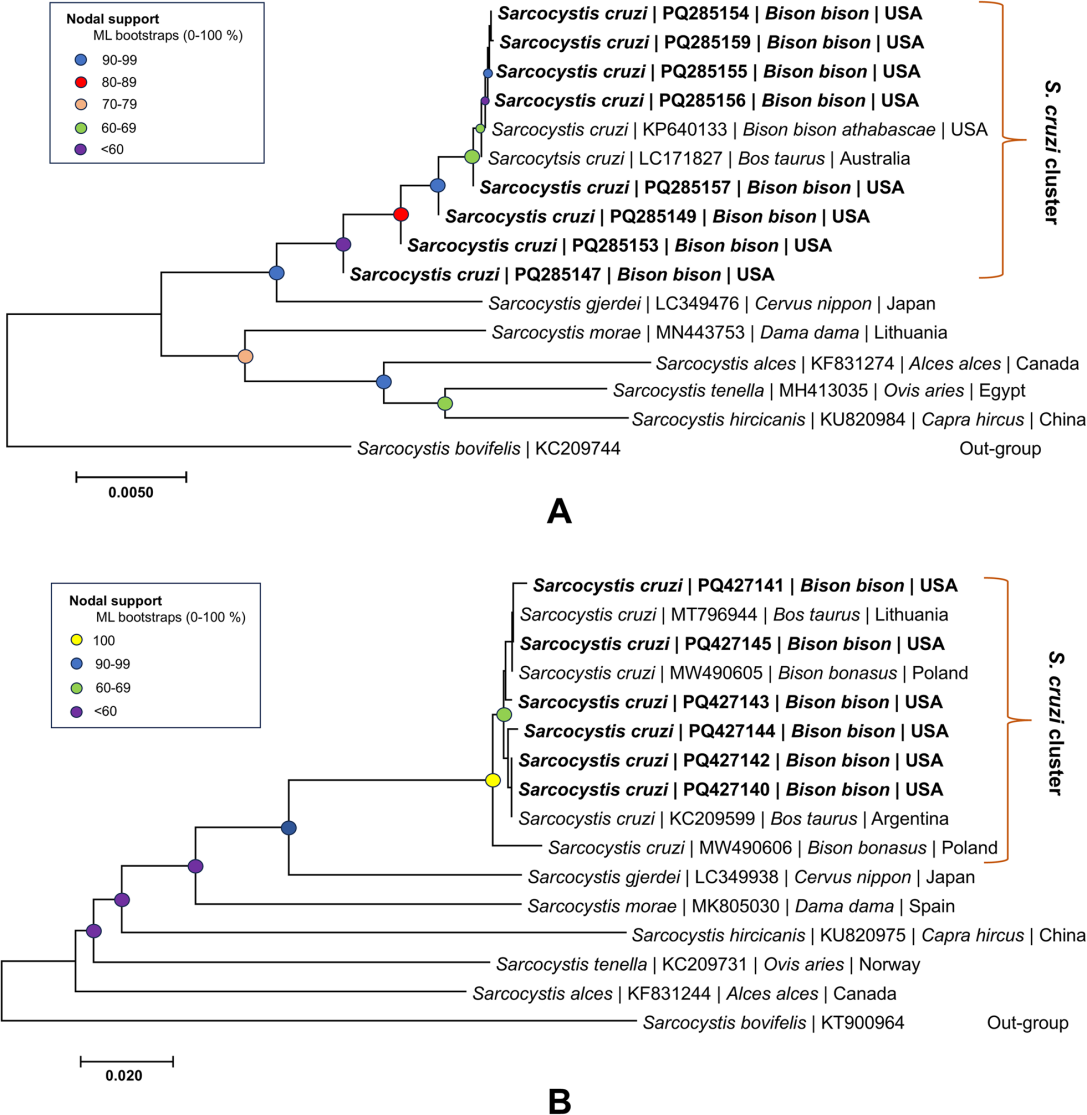
Phylogenetic tree reconstructed by maximum likelihood showing the phylogenetic position of *Sarcocystis cruzi* identified in *Bison bison* tongue samples using the gene markers *18S* ribosomal RNA gene (*18S* rRNA) (**A**) and cytochrome* c* oxidase subunit 1 (*cox1*) gene (**B**). The “*S. cruzi* cluster” is highlighted with orange brackets in both the trees. Species names shown in bold are the those detected during the present study

In the phylogenetic tree we constructed based on partial *18S* rRNA and *cox1* genes (Fig. 3), the sequences obtained during the study formed a strongly supported cluster with sequences attributed to *S. cruzi* (for *18S*: accession no. KP640133, LC171827; for *cox1*: acccession no. MT796944, MW490605-6, KC209599). In Fig. 3, The *“S. cruzi* cluster” has been highlighted with brackets in the tree. *Sarcocystis gjerdei* also formed the extended cluster with *S. cruzi*. The other* Sarcocystis* species forming the basal clade are *S. morae*, *S. alces*, *S. tenella* and *S. hircicanis*.

The tree agrees with previous studies on *S. cruzi* wherein the molecular analysis and phylogenetic position, prevalence and occurrence have been discussed in the cattle and bison (European and wood bison) [20, 21, 23, 24].

Interestingly, there was a slight deviation in the clustering of these *Sarcocystis* species infecting closely related hosts, which is evident from the different range of consensus and branch length, even in the closely related species. Despite these variations, the relationships between species within each clade remained relatively consistent, irrespective of the branching order of the major clades involving different isolates of *S. cruzi,* showing the close evolutionary relationship between the species.

### Unidentified sarcocysts

Among the eight species of *Sarcocystis* in cattle, *S. cruzi* and *S. heydorni* have sarcocysts with thin walls (< 1 µm) whereas the cyst walls of the other six species (*S. hominis, S. bovifelis, S. bovini*, *S. sigmoideus, S. hirsuta*, and *S. rommeli)* are thicker than 3 µm and also appear striated under the light microscope [8]. We did not detect any thick-walled sarcocysts in the present study. Rather, in two bison samples (#94 and #108) we detected cysts with walls around 2 µm thick that were not striated (Fig. 1b, d), which we were unable to identify. These latter cysts were present together with thin-walled *S. cruzi* sarcocysts in these samples.

## Conclusions

More research is needed to understand whether species other than *S. cruzi,* present in cattle, also occur in bison. The high prevalence of *S. cruzi* in the American bison tongue samples (95.5%) studied indicates the presence of contaminated fecal material from carnivores in the open range of cattle within the same area. The results of the present study show that range management may reduce livestock to exposure.

## Supplementary Information

Additional file 1: Figure S1

Additional file 2: Figure S2.

Additional file 3: Table S1.

## Data Availability

No datasets were generated or analysed during the current study.

## Abbreviations

*cox1*: Cytochrome* c* oxidase subunit 1
CT: Cycle threshold
HE: Hematoxylin and eosin
LM: Light microscopy
NT: Near threatened
*18S* rRNA: *18S* ribosomal RNA
*28S* rRNA: *28S* ribosomal RNA
qPCR: Quantitative PCR
USDA: U.S. States Department of Agriculture
