# Supplementary figures and images for: High, but variable prevalence of Sarcocystis cruzi infections in farm-raised American bison (Bison bison) beef destined for human consumption
Source: Parasit Vectors. 2025 Feb 1;18:35. doi: 10.1186/s13071-025-06660-y (PMC11787736; doi:10.1186/s13071-025-06660-y)

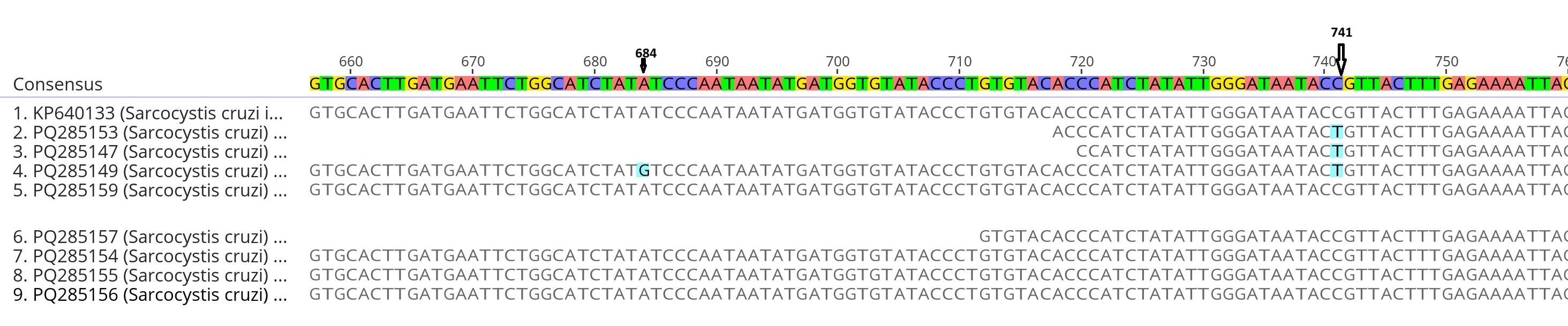

Supplement: Supplementary file 1 — Additional file 1: Figure S1 [file 13071_2025_6660_MOESM1_ESM.jpg]

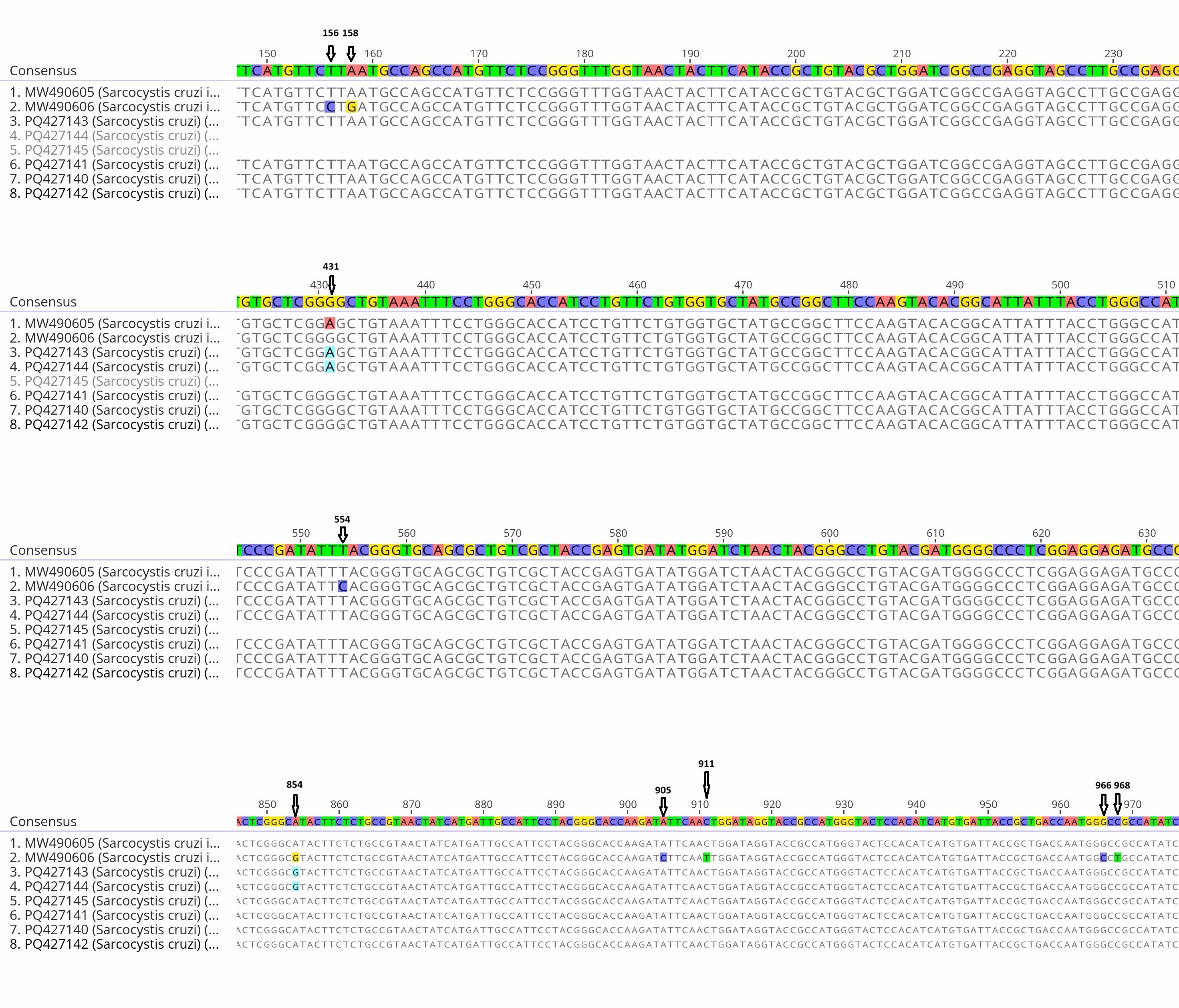

Supplement: Supplementary file 2 — Additional file 2: Figure S2. [file 13071_2025_6660_MOESM2_ESM.jpg]
