# Supplementary material for: High, but variable prevalence of Sarcocystis cruzi infections in farm-raised American bison (Bison bison) beef destined for human consumption
Source: Parasit Vectors. 2025 Feb 1;18:35. doi: 10.1186/s13071-025-06660-y (PMC11787736; doi:10.1186/s13071-025-06660-y)
Supplement: Supplementary file 3 — Additional file 3: Table S1. [file 13071_2025_6660_MOESM3_ESM.docx]

**Supplementary Table 1**. DNA concentration measured by Nanodrop and cycle threshold (CT) values of qPCR for *Sarcocystis cruzi* *18S* gene marker in comparison with microscopy results of the 200 samples analyzed.

| **Sample No.** | **Nanodrop Value (ng/μL)** | **CT Value (20ng DNA)** | **REPEATS - CT Value (60ng DNA)** | **Direct Smears** | **No. of cysts found in H&E Slides** |  |
| --- | --- | --- | --- | --- | --- | --- |
|  |  |  |  |  |  |  |
|  |  |  |  |  |  |  |
| 1 | 763 | 13.715 |  | POSITIVE - THIN WALL CYST | 18 |  |
| 2 | 1008 | 28.426 |  | POSITIVE - THIN WALL CYST | 8 |  |
| 3 | 51 | 34.441 |  | POSITIVE - THIN WALL CYST | 15 |  |
| 4 | 83 | 28.096 |  | POSITIVE - THIN WALL CYST | 4 |  |
| 5 | 22 | 15.567 | 27.606 | POSITIVE - THIN WALL CYST | 10 |  |
| 6 | 1907 | 32.162 |  | POSITIVE - THIN WALL CYST | 10 |  |
| 7 | 2156 | 29.177 |  | POSITIVE - THIN WALL CYST | 3 |  |
| 8 | 185 | NEGATIVE | NEGATIVE | NEGATIVE | 6 |  |
| 9 | 229 | 29.843 |  | POSITIVE - THIN WALL CYST | 53 |  |
| 10 | 32 | 29.595 |  | POSITIVE - THIN WALL CYST | 5 |  |
| 11 | 347 | 36.675 | 33.387 | NEGATIVE | 2 |  |
| 12 | 2225 | 32.659 |  | NEGATIVE | 8 |  |
| 13 | 7.1 | 27.773 |  | POSITIVE - THIN WALL CYST | 5 |  |
| 14 | 9.5 | 27.236 |  | POSITIVE - THIN WALL CYST | 44 |  |
| 15 | 131 | 33.620 |  | NEGATIVE | Negative |  |
| 16 | 36 | 29.395 |  | POSITIVE - THIN WALL CYST | 3 |  |
| 17 | 450 | 32.359 |  | NEGATIVE | 15 |  |
| 18 | 45 | 29.778 |  | POSITIVE - THIN WALL CYST | Negative |  |
| 19 | 10.8 | 27.333 |  | POSITIVE - THIN WALL CYST | 11 |  |
| 20 | 57 | 31.073 |  | POSITIVE - THIN WALL CYST | 13 |  |
| 21 | 194 | 30.849 |  | POSITIVE - THIN WALL CYST | 16 |  |
| 22 | 247 | 33.173 |  | POSITIVE - THIN WALL CYST | Negative |  |
| 23 | 99.5 | 33.972 |  | POSITIVE - THIN WALL CYST | 12 |  |
| 24 | 291 | 29.162 |  | POSITIVE - THIN WALL CYST | 45 |  |
| 25 | 145 | 32.099 |  | POSITIVE - THIN WALL CYST | 2 |  |
| 26 | 183 | 29.250 |  | NEGATIVE | 1 |  |
| 27 | 133 | 31.565 |  | POSITIVE - THIN WALL CYST | 12 |  |
| 28 | 460 | 37.815 | 36.979 | NEGATIVE | Negative |  |
| 29 | 515 | 35.173 | 32.566 | POSITIVE - THIN WALL CYST | Negative |  |
| 30 | 439 | 33.626 |  | NEGATIVE | 2 |  |
| 31 | 445 | 28.428 |  | POSITIVE - THIN WALL CYST | 15 |  |
| 32 | 503 | 32.866 |  | POSITIVE - THIN WALL CYST | 8 |  |
| 33 | 650 | 34.835 |  | POSITIVE - THIN WALL CYST | 43 |  |
| 34 | 407 | 29.745 |  | POSITIVE - THIN WALL CYST | 29 |  |
| 35 | 200 | 31.701 |  | POSITIVE - THIN WALL CYST | 5 |  |
| 36 | 80 | 32.003 |  | POSITIVE - THIN WALL CYST | 25 |  |
| 37 | 217 | 30.671 |  | NEGATIVE | 7 |  |
| 38 | 66 | 35.934 | 36.012 | NEGATIVE | 9 |  |
| 39 | 284 | 27.724 |  | POSITIVE - THIN WALL CYST | 20 |  |
| 40 | 134 | 29.536 |  | POSITIVE - THIN WALL CYST | 31 |  |
| 41 | 100 | 29.323 |  | POSITIVE - THIN WALL CYST | 17 |  |
| 42 | 284 | 29.442 |  | POSITIVE - THIN WALL CYST | 3 |  |
| 43 | 1.88 | 26.282 |  | POSITIVE - THIN WALL CYST | 50 |  |
| 44 | 197 | 24.084 |  | POSITIVE - THIN WALL CYST | 20 |  |
| 45 | 296 | 32.467 |  | NEGATIVE | 1 |  |
| 46 | 39 | 28.809 |  | POSITIVE - THIN WALL CYST | 3 |  |
| 47 | 118 | 27.369 |  | POSITIVE - THIN WALL CYST | 27 |  |
| 48 | 126 | 26.848 |  | POSITIVE - THIN WALL CYST | 51 |  |
| 49 | 203.7 | 30.099 |  | NEGATIVE | Negative |  |
| 50 | 11.3 | 28.175 |  | POSITIVE - THIN WALL CYST | 6 |  |
| 51 | 29.4 | 30.567 |  | POSITIVE - THIN WALL CYST | 7 |  |
| 52 | 44.5 | 34.248 |  | POSITIVE - THIN WALL CYST | 9 |  |
| 53 | 18.6 | 33.802 |  | POSITIVE - THIN WALL CYST | 10 |  |
| 54 | 18.5 | 35.184 | 30.697 | POSITIVE - THIN WALL CYST | 2 |  |
| 55 | 19.5 | 31.465 |  | POSITIVE - THIN WALL CYST | 27 |  |
| 56 | 43.5 | 30.532 |  | POSITIVE - THIN WALL CYST | 5 |  |
| 57 | 33 | 30.788 |  | POSITIVE - THIN WALL CYST | 17 |  |
| 58 | 62.1 | 31.023 |  | POSITIVE - THIN WALL CYST | 4 |  |
| 59 | 45.5 | 29.211 |  | POSITIVE - THIN WALL CYST | 31 |  |
| 60 | 47 | 33.147 |  | POSITIVE - THIN WALL CYST | 6 |  |
| 61 | 34.6 | 35.101 | 28.076 | POSITIVE - THIN WALL CYST | 4 |  |
| 62 | 44.5 | 29.284 |  | POSITIVE - THIN WALL CYST | 4 |  |
| 63 | 54.9 | 32.154 |  | POSITIVE - THIN WALL CYST | 2 |  |
| 64 | 102.5 | 30.999 |  | POSITIVE - THIN WALL CYST | 31 |  |
| 65 | 76.5 | 29.961 |  | POSITIVE - THIN WALL CYST | 14 |  |
| 66 | 52.8 | 31.104 |  | POSITIVE - THIN WALL CYST | 5 |  |
| 67 | 59.6 | 31.339 |  | POSITIVE - THIN WALL CYST | 16 |  |
| 68 | 96.5 | 33.602 |  | POSITIVE - THIN WALL CYST | 2 |  |
| 69 | 51.3 | 29.297 |  | POSITIVE - THIN WALL CYST | 5 |  |
| 70 | 63.6 | 33.757 |  | POSITIVE - THIN WALL CYST | 13 |  |
| 71 | 185 | 27.883 |  | POSITIVE - THIN WALL CYST | 5 |  |
| 72 | 237 | 27.168 |  | POSITIVE - THIN WALL CYST | 11 |  |
| 73 | 115 | 38.715 | 36.875 | NEGATIVE | Negative |  |
| 74 | 129 | 28.681 |  | POSITIVE - THIN WALL CYST | 20 |  |
| 75 | 119 | 24.833 |  | POSITIVE - THIN WALL CYST | 88 |  |
| 76 | 56 | 31.341 |  | POSITIVE - THIN WALL CYST | 6 |  |
| 77 | 22 | 29.157 |  | POSITIVE - THIN WALL CYST | 4 |  |
| 78 | 220 | 27.237 |  | POSITIVE - THIN WALL CYST | 68 |  |
| 79 | 79 | 27.750 |  | POSITIVE - THIN WALL CYST | 65 |  |
| 80 | 16 | 26.093 |  | POSITIVE - THIN WALL CYST | 34 |  |
| 81 | 223 | 25.843 |  | POSITIVE - THIN WALL CYST | 24 |  |
| 82 | 131.7 | 29.415 |  | POSITIVE - THIN WALL CYST | 31 |  |
| 83 | 163.2 | 27.416 |  | POSITIVE - THIN WALL CYST | 3 |  |
| 84 | 146.1 | 30.381 |  | POSITIVE - THIN WALL CYST | 4 |  |
| 85 | 156.2 | 28.492 |  | POSITIVE - THIN WALL CYST | 17 |  |
| 86 | 41.7 | 32.854 |  | POSITIVE - THIN WALL CYST | SLIDE A=5, SLIDE B=15 |  |
| 87 | 27.8 | 33.152 |  | POSITIVE - THIN WALL CYST | SLIDE A=1, SLIDE B=5 |  |
| 88 | 177.6 | 26.951 |  | POSITIVE - THIN WALL CYST | SLIDE A=104, SLIDE B=67 |  |
| 89 | 71.6 | 32.158 |  | POSITIVE - THIN WALL CYST | SLIDE A=1, SLIDE B=3 |  |
| 90 | 26.2 | 34.387 |  | NEGATIVE | Negative |  |
| 91 | 62.2/63 | 27.719 |  | POSITIVE - THIN WALL CYST | SLIDE A=13, SLIDE B=16 |  |
| 92 | 34.8 | 31.777 |  | POSITIVE - THIN WALL CYST | SLIDE A=16, SLIDE B=14 |  |
| 93 | 57.1 | 27.427 |  | POSITIVE - THIN WALL CYST | SLIDE A=16, SLIDE B=21 |  |
| 94 | 103.9 | 21.729 |  | POSITIVE - THIN WALL CYST | SLIDE A=82, SLIDE B=64 |  |
| 95 | 46.4 | 27.404 |  | POSITIVE - THIN WALL CYST | SLIDE A=33, SLIDE B=39 |  |
| 96 | 85.6 | 29.397 |  | POSITIVE - THIN WALL CYST | SLIDE A=11, SLIDE B=4 |  |
| 97 | 25.5 | 35.502 | 33.299 | NEGATIVE | SLIDE A=NEG, SLIDE B=1 |  |
| 98 | 24.5 | 32.935 |  | NEGATIVE | SLIDE A=NEG, SLIDE B=1 |  |
| 99 | 112.9 | 32.878 |  | NEGATIVE | Negative |  |
| 100 | 42.3 | 29.896 |  | POSITIVE - THIN WALL CYST | SLIDE A=8, SLIDE B=14 |  |
| 101 | 155.1 | 29.947 |  | POSITIVE - THIN WALL CYST | 101 |  |
| 102 | 122.6 | 27.523 |  | POSITIVE - THIN WALL CYST | 63 |  |
| 103 | 232.5 | 25.867 |  | POSITIVE - THIN WALL CYST | 34 |  |
| 104 | 208.2 | 29.308 |  | POSITIVE - THIN WALL CYST | 11 |  |
| 105 | 100.7 | 29.862 |  | POSITIVE - THIN WALL CYST | 25 |  |
| 106 | 237.9 | 27.795 |  | POSITIVE - THIN WALL CYST | 3 |  |
| 107 | 175.3 | 27.161 |  | POSITIVE - THIN WALL CYST | 27 |  |
| 108 | 278.4 | 24.599 |  | POSITIVE - THIN WALL CYST | 260 |  |
| 109 | 74.5 | 33.170 |  | POSITIVE - THIN WALL CYST | SLIDE A=5, SLIDE B=4 |  |
| 110 | 114.2 | 29.167 |  | POSITIVE - THIN WALL CYST | SIDE A=84, SLIDE B=39 |  |
| 111 | 130.1 | 34.771 |  | POSITIVE - THIN WALL CYST | SLIDE A=1, SLIDE B=NEG |  |
| 112 | 161.9 | 31.176 |  | POSITIVE - THIN WALL CYST | SLIDE A=24, SLIDE B=35 |  |
| 113 | 302.2 | 30.721 |  | POSITIVE - THIN WALL CYST | SLIDE A=23, SLIDE B=7 |  |
| 114 | 119 | 32.303 |  | POSITIVE - THIN WALL CYST | SLIDE A=8, SLIDE B=10 |  |
| 115 | 108.6 | 34.988 |  | NEGATIVE | SLIDE A=1, SLIDE B=NEG |  |
| 116 | 107.3 | 30.874 |  | POSITIVE - THIN WALL CYST | SLIDE A=7, SLIDE B=9 |  |
| 117 | 84.5 | 32.105 |  | POSITIVE - THIN WALL CYST | 1 |  |
| 118 | 27.1 | 33.816 |  | POSITIVE - THIN WALL CYST | 6 |  |
| 119 | 54.2 | 31.255 |  | POSITIVE - THIN WALL CYST | 11 |  |
| 120 | 74.7 | NEGATIVE | 35.994 | NEGATIVE | Negative |  |
| 121 | 166.1 | 30.667 |  | POSITIVE - THIN WALL CYST | 43 |  |
| 122 | 173.6 | 29.549 |  | POSITIVE - THIN WALL CYST | 98 |  |
| 123 | 65.8 | 29.293 |  | POSITIVE - THIN WALL CYST | 2 |  |
| 124 | 98.5 | 33.280 |  | POSITIVE - THIN WALL CYST | Negative |  |
| 125 | 175 | 32.999 |  | POSITIVE - THIN WALL CYST | 32 |  |
| 126 | 171.1 | 31.404 |  | POSITIVE - THIN WALL CYST | 8 |  |
| 127 | 125.9 | 37.687 | 36.589 | POSITIVE - THIN WALL CYST | 3 |  |
| 128 | 115.3 | 27.130 |  | POSITIVE - THIN WALL CYST | 121 |  |
| 129 | 178.8 | 29.226 |  | POSITIVE - THIN WALL CYST | 4 |  |
| 130 | 167.1 | 30.551 |  | POSITIVE - THIN WALL CYST | 16 |  |
| 131 | 12.5 | 32.922 |  | POSITIVE - THIN WALL CYST | 7 |  |
| 132 | 80.6 | 33.991 |  | POSITIVE - THIN WALL CYST | 1 |  |
| 133 | 9.7 | 31.715 |  | POSITIVE - THIN WALL CYST | 27 |  |
| 134 | 15.3 | 32.467 |  | POSITIVE - THIN WALL CYST | 8 |  |
| 135 | 38.4 | 33.917 |  | POSITIVE - THIN WALL CYST | 12 |  |
| 136 | 56.2 | 33.913 |  | POSITIVE - THIN WALL CYST | 10 |  |
| 137 | 3.2 | 32.556 |  | POSITIVE - THIN WALL CYST | 47 |  |
| 138 | 77.7 | 38.056 | 34.590 | NEGATIVE | Negative |  |
| 139 | 27.4 | 32.025 |  | POSITIVE - THIN WALL CYST | 20 |  |
| 140 | 58.6 | 33.470 |  | POSITIVE - THIN WALL CYST | 20 |  |
| 141 | 77.1 | 30.272 |  | POSITIVE - THIN WALL CYST | 24 |  |
| 142 | 120.7 | 32.322 |  | POSITIVE - THIN WALL CYST | 9 |  |
| 143 | 69 | 30.313 |  | POSITIVE - THIN WALL CYST | 9 |  |
| 144 | 80.6 | 31.691 |  | POSITIVE - THIN WALL CYST | 10 |  |
| 145 | 57.5 | 32.485 |  | POSITIVE - THIN WALL CYST | 13 |  |
| 146 | 88.6 | 34.874 |  | NEGATIVE | 2 |  |
| 147 | 141.2 | 28.909 |  | POSITIVE - THIN WALL CYST | 15 |  |
| 148 | 23.5 | 27.809 |  | POSITIVE - THIN WALL CYST | 22 |  |
| 149 | 115.6 | 30.945 |  | POSITIVE - THIN WALL CYST | 10 |  |
| 150 | 30.1 | 31.790 |  | POSITIVE - THIN WALL CYST | 13 |  |
| 151 | 156 | 32.987 |  | POSITIVE - THIN WALL CYST | 20 |  |
| 152 | 81.8 | 32.346 |  | POSITIVE - THIN WALL CYST | 3 |  |
| 153 | 128 | 31.243 |  | POSITIVE - THIN WALL CYST | 31 |  |
| 154 | 111.9 | 32.470 |  | POSITIVE - THIN WALL CYST | 6 |  |
| 155 | 130.6 | 33.399 |  | POSITIVE - THIN WALL CYST | 14 |  |
| 156 | 177.6 | 31.109 |  | POSITIVE - THIN WALL CYST | 10 |  |
| 157 | 172.9 | 32.645 |  | POSITIVE - THIN WALL CYST | 10 |  |
| 158 | 209.2 | 31.488 |  | POSITIVE - THIN WALL CYST | 21 |  |
| 159 | 208.1 | 29.903 |  | POSITIVE - THIN WALL CYST | 27 |  |
| 160 | 28 | 33.420 |  | POSITIVE - THIN WALL CYST | 11 |  |
| 161 | 14 | 31.843 |  | POSITIVE - THIN WALL CYST | 37 |  |
| 162 | 25.1 | 33.858 |  | POSITIVE - THIN WALL CYST | 19 |  |
| 163 | 27.5 | 32.115 |  | POSITIVE - THIN WALL CYST | 9 |  |
| 164 | 14.3 | 33.281 |  | POSITIVE - THIN WALL CYST | 15 |  |
| 165 | 35.2 | 33.861 |  | POSITIVE - THIN WALL CYST | 40 |  |
| 166 | 12.4 | 33.521 |  | POSITIVE - THIN WALL CYST | 14 |  |
| 167 | 13 | 31.197 |  | POSITIVE - THIN WALL CYST | 29 |  |
| 168 | 33.8 | 33.452 |  | POSITIVE - THIN WALL CYST | 14 |  |
| 169 | 64.4 | 32.754 |  | POSITIVE - THIN WALL CYST | 36 |  |
| 170 | 36 | 32.429 |  | POSITIVE - THIN WALL CYST | 14 |  |
| 171 | 19.7 | 32.961 |  | POSITIVE - THIN WALL CYST | 14 |  |
| 172 | 26.6 | 30.263 |  | POSITIVE - THIN WALL CYST | 8 |  |
| 173 | 18.8 | 36.074 | 30.175 | POSITIVE - THIN WALL CYST | 1 |  |
| 174 | 13.9 | 34.562 |  | POSITIVE - THIN WALL CYST | 2 |  |
| 175 | 38.8 | 31.860 |  | POSITIVE - THIN WALL CYST | 24 |  |
| 176 | 13.9 | 32.805 |  | POSITIVE - THIN WALL CYST | 15 |  |
| 177 | 51.6 | 32.614 |  | POSITIVE - THIN WALL CYST | 19 |  |
| 178 | 87.2 | 39.609 | NEGATIVE | NEGATIVE | Negative |  |
| 179 | 179.5 | 36.809 | 34.459 | NEGATIVE | Negative |  |
| 180 | 74.8 | 20.032 | NEGATIVE | NEGATIVE | Negative |  |
| 181 | 58.7 | NEGATIVE | NEGATIVE | NEGATIVE | Negative |  |
| 182 | 124.6 | 24.785 | NEGATIVE | NEGATIVE | Negative |  |
| 183 | 52.9 | NEGATIVE | NEGATIVE | NEGATIVE | Negative |  |
| 184 | 140 | NEGATIVE | NEGATIVE | NEGATIVE | Negative |  |
| 185 | 90.8 | 37.125 | 31.923 | NEGATIVE | Negative |  |
| 186 | 38.5 | NEGATIVE | NEGATIVE | NEGATIVE | Negative |  |
| 187 | 10 |  | 1.000 | NEGATIVE | Negative |  |
| 188 | 30.5 | NEGATIVE | NEGATIVE | NEGATIVE | Negative |  |
| 189 | 14.4 | NEGATIVE | NEGATIVE | NEGATIVE | Negative |  |
| 190 | 22.4 | 37.375 | 31.941 | NEGATIVE | Negative |  |
| 191 | 18.8 | NEGATIVE | 34.259 | NEGATIVE | 1 |  |
| 192 | 74.5 | 38.354 | NEGATIVE | NEGATIVE | Negative |  |
| 193 | 98.5 | 26.780 | NEGATIVE | NEGATIVE | Negative |  |
| 194 | 7.5 | 38.371 | 37.528 | NEGATIVE | Negative |  |
| 195 | 107.2 | 39.160 | 35.387 | NEGATIVE | Negative |  |
| 196 | 68.6 | 37.833 | 33.457 | NEGATIVE | Negative |  |
| 197 | 123.3 | NEGATIVE | NEGATIVE | NEGATIVE | Negative |  |
| 198 | 6.8 | NEGATIVE | NEGATIVE | NEGATIVE | Negative |  |
| 199 | 55 | 36.996 | 32.356 | POSITIVE - THIN WALL CYST | 1 |  |
| 200 | 13.9 | 38.231 | 31.751 | NEGATIVE | Negative |  |

Gray cell=Not done, H&E=Hematoxylin and Eosin Stain
